# Linguistic and Cognitive Effects of Bilingualism with Regional Minority Languages: A Study of Sardinian–Italian Adult Speakers

**DOI:** 10.3389/fpsyg.2017.01907

**Published:** 2017-11-01

**Authors:** Maria Garraffa, Mateo Obregon, Antonella Sorace

**Affiliations:** ^1^Department of Psychology, Heriot-Watt University, Edinburgh, United Kingdom; ^2^Department of Linguistics and English Language, The University of Edinburgh, Edinburgh, United Kingdom

**Keywords:** minority languages, sentences processing, working memory, bilingualism, relative clauses, Sardinian

## Abstract

This study explores the effects of bilingualism in Sardinian as a regional minority language on the linguistic competence in Italian as the dominant language and on non-linguistic cognitive abilities. Sardinian/Italian adult speakers and monolingual Italian speakers living in the same geographical area of Sardinia were compared in two kinds of tasks: (a) verbal and non-verbal cognitive tasks targeting working memory and attentional control and (b) tasks of linguistic abilities in Italian focused on the comprehension of sentences differing in grammatical complexity. Although no difference was found between bilinguals and monolinguals in the cognitive control of attention, bilinguals performed better on working memory tasks. Bilinguals with lower formal education were found to be faster at comprehension of one type of complex sentence (center embedded object relative clauses). In contrast, bilinguals and monolinguals with higher education showed comparable slower processing of complex sentences. These results show that the effects of bilingualism are modulated by type of language experience and education background: positive effects of active bilingualism on the dominant language are visible in bilinguals with lower education, whereas the effects of higher literacy in Italian obliterate those of active bilingualism in bilinguals and monolinguals with higher education.

## Introduction

One of the under-explored topics in current research on bilingualism is the effect of language status and prestige on the linguistic and cognitive characteristics of bilingual competence. The language experience of speakers growing up with more than one language is in fact subject to considerable variation due to different environmental factors, including the contexts of use and the registers adopted for different languages: for example, minority languages are often used in more informal contexts while dominant languages are used in more formal circumstances, including schooling and education. Minority languages therefore provide an ideal ground to explore the influence of these variables. At the same time, minority languages are typically spoken by natives of particular regions: this offers the opportunity to control for socio-economic and cultural differences, which typically characterize other types of bilingual experience, for example bilingualism introduced by migration.

In this paper we focus on the island of Sardinia, where bilingualism with Sardinian – the local minority language – and Italian is the norm, especially in the central areas. We compare bilingual Sardinian–Italian adults with monolingual Italian adults living in the same area, with a twofold aim. First, we address the widespread view that Sardinian undermines competence in Italian, which is often perceived as a negative consequence of bilingualism in these two languages. In testing linguistic competence in Italian in bilingual speakers, the effects of education need to be controlled, since it has been reported in sociolinguistic research that speakers with lower education are more active users of the minority language (Oppo, 2007). Second, we look at some of the general cognitive abilities that have previously been found to be enhanced by the bilingual experience (see section “Cognitive Effects of Bilingualism in Regional Minority Languages”): specifically, we compare bilingual and monolinguals on standard tests of working memory and cognitive control.

Focusing on adult speakers is important for several reasons. Adults who are bilingual with minority languages typically use the majority language in the workplace and in most daily activities, and speak the minority language in a much more restricted range of contexts. This is the age where decisions about intergenerational transmission of the minority language are made, which are increasingly in favor of not speaking it to children due to a perceived lack of usefulness. Adult competence in minority languages – whether native or non-native – has been argued to play a fundamental role in reversing this shift. If native speakers of childbearing age who are fully confident in using the language with their children are decreasing, “new” speakers of minority languages can play a role in re-establishing intergenerational transmission (Fishman, 2001; O’Rourke and Pujolar, 2015).

The incentives for speaking a minority language to children might include the possible benefits of bilingualism gained through this particular bilingual experience, if these are supported by research.

In the next sections we will briefly summarize some recent findings reported in studies of bilingualism with minority languages, with an emphasis on grammatical competence and general cognition. We will then turn to some background information on the status of Sardinian, before motivating our research questions and presenting the results of our study.

## Cognitive Effects of Bilingualism in Regional Minority Languages

A large number of studies in the last 15 years report positive effects of bilingualism on mental flexibility, specifically in terms of enhancement of the cognitive abilities referred to as “executive functions” (Bialystok, 2009; Baum and Titone, 2014; Costa and Sebastian-Galles, 2014 for overviews). More recently, these findings have been questioned by studies reporting no “bilingual advantage” in these cognitive domains (Paap and Greenberg, 2013). The picture emerging from the limited number of studies exploring cognitive abilities in bilingualism with minority languages is also inconsistent. While no bilingual advantage in executive functions was found in studies of Welsh–English bilinguals (Gathercole et al., 2014) and Basque–Spanish bilinguals (Duñabeitia et al., 2014), other studies show an advantage for bilingual speakers of minority languages such as Scottish Gaelic (Lauchlan et al., 2013), Sardinian [Lauchlan et al. (2013) on adults; Garraffa et al. (2015) on children], and Cypriot Greek (Antoniou et al., 2016). While the evidence based on these studies is too scarce to allow generalizations, it is possible that the type of bilingual experience associated with different minority languages may lead to different (or null) effects on cognitive abilities. For instance, Costa et al. (2009) proposed that speakers with highly separated and predictable domains of use for each language – thus with a low level of switching required – may not show advantages. Similarly, Prior and Gollan (2011) suggest that an advantage in cognitive control may arise only in bilinguals who frequently switch between languages. The typological relatedness of language pairs may also be relevant [Costa et al. (2009); see Grohmann (2014) and Kyriakos et al. (2016) on “language proximity” as an important factor for simultaneous child bilingualism]. Finally, the presence or absence of minority languages in education program and their availability as a medium of instruction may lead to a wider range of uses and enhance possible effects outside the language domain.

## Linguistic Abilities and Knowledge of Grammar in Bilingualism

Many studies on bilingual children have reported a bilingual advantage in tasks related to grammatical knowledge, such as grammaticality judgments of sentences and correction of syntactically incorrect sentences (Galambos and Goldin-Meadow, 1990). This bilingual advantage on metalinguistic tasks, especially in the context of detecting semantic anomalies, was replicated across different languages [e.g., Ricciardelli (1992) with Italian–English; Cromdal (1999) with Swedish–English see Barak and Bialystok (2016) for an overview]. As far as bilingual adults, ERPs study by Moreno et al. (2010) recorded markers related to semantic (N400) and syntactic (eLAN, LAN, and P600) analyses during reading and during a sentence judgment task. They found that bilingual experience has an impact on sentence processing and this is more visible in judgment tasks that require selective attention compared to acceptability tasks, based primarily on syntactic knowledge.

Previous research described the role of enhanced as well as impaired short-term memory for comprehension of relative clauses, in particular for comprehension of object relatives such as (2) and (4) (Lauro et al., 2010; Papagno et al., 2012)^1^. Several accounts suggest that comprehension of complex sentences is facilitated by working memory (e.g., Gordon and Olson, 1998).

(1) Subject peripheral: The grandfather pushes *the dog* that <the dog> bites the cat.
(2) Object peripheral: The mother looks at *the dog* that the **boy** chases <the dog>.
(3) Subject center embedded: *The boy* that is watching the cat <the boy> is drinking milk.
(4) Object center embedded: *The boy* that the **cat** is chasing <the boy> is looking at the girl.

In this study we focus on comprehension of complex sentences in Italian and the possible relationship between linguistic and general cognitive factors. Specifically, the aim of the study is to test whether the bilingual experience with Sardinian could affect the processing of complex sentences in the dominant language. The range of sentence types investigated included active and passive sentences, coordinated sentences, and relative clauses varying in complexity. These sentences are part of the *Comprendo* standardized comprehension test (Cecchetto et al., 2012); see the section “Materials and Methods” Sardinian has both similar and different constructions from Italian; the main difference between the two languages is the rare use of the passive form and of center embedded object relatives in Sardinian. All sentence types included in the study and corresponding Sardinian translations are summarized in **Table 1**.

**Table 1:** Sentence structure types tested in the *Comprendo* test of Italian and corresponding Sardinian translations.

Type	Italian example	Sardinian translation
Active	Il cane morde il gatto	Su cane mossigat (a) sa gato
	*The dog bites the cat*	*The dog bites the cat*
Dative	La mamma dà la torta al bambino	Sa mamma li dat su durce a su pitzinneddu
	*The mother gives the cake to the boy*	*The mother to-him gives the cake to the boy*
Passive	Il bambino viene inseguito dal cane	Su pitzinneddu est pressighidu da esu cane
	*The boy is chased by the dog*	*The boy is chased by the dog*
		Su pitzinneddu lu pressighit su cane
		*The boy him chases the dog*
Peripheral subject relative	Il nonno spinge il cane che morde il gatto	Su mannoi ispinghet su cane chi mossigat sa gato
	*The grandfather pushes the dog that bites the cat*	*The grandfather pushes the dog that bites the cat*
Peripheral object relative	La mamma guarda il cane che il bambino insegue	Sa mamma abbaidat su cane chi su pitzinneddu pressighit
	*The mother looks at the dog that the boy chases*	*The mother looks at the dog that the boy chases*
Center embedded subject relative	Il bambino che sta guardando il gatto beve il latte	Su pitzinneddu chi est pompiande sa gato buffat su latte
	*The boy that is watching the cat is drinking milk*	*The boy that is watching the cat is drinking milk*
Center-embedded object relative	Il ragazzo che il cane insegue sta guardando la ragazza	Su pitzinneddu chi l’est sighinde su cane est pompiande sa picinna
	*The boy that the cat is chasing is looking at the girl*	*The boy that he is chased by the dog is looking at the girl*
Object coordination	Il bambino insegue il cane e il gatto	Su pitzinneddu pressighit su cane e sa gato
	*The boy chases the dog and the cat*	*The boy chases the dog and the cat*
Verb coordination	Il bambino guarda il cane e accarezza il gatto	Su pilosu pompiat su cane e caringiat sa gato
	*The boy looks at the dog and strokes the cat*	*The boy looks at the dog and strokes the cat*
Sentence coordination	Il bambino guarda il gatto e la mamma accarezza il cane	Su pitzinneddu pompiat sa gato e sa mamma caringiat su cane
	*The boy looks at the cat and the mum strokes the dog*	*The boy looks at the cat and the mum strokes the dog*


A cognitively based model would predict differences between bilinguals and monolinguals in the processing of complex sentences, such as object relative clauses, due to an enhanced memory capacity in bilinguals (Bialystok, 2007). A linguistically based model would predict bilingual–monolingual differences in processing due to a different grammatical representation in bilinguals, compared to monolingual speakers (Belletti and Guasti, 2015). Both models predict a different performance for complex sentences in active bilinguals compared to speakers who are not actively using the minority language.

Evidence for a better performance on object relative production in bilingual speakers was reported by Belletti and Guasti (2015) in a group of beginners L2 learners compared to advanced L2 learners and in the previously mentioned study on Sardinian/Italian bilingual children (Garraffa et al., 2015), which found that comprehension of object relative in Italian improved significantly more in bilingual children compared to monolinguals^2^.

Another aspect tested in the present study is the impact of education, in particular the combined effect of high competence in the dominant language and reduced use of the minority language (see description of participants below). Dubrowska and Street (2006) in a study on comprehension of passive sentences by native and non-native English speakers reported a better performance of the less-educated non-native group compared to the native group matched for education, although memory and cognitive abilities were not controlled for. The authors suggested that processing more complex sentences, such as passives, depends on metalinguistic skills and this metalinguistic competence could be enhanced in L2 learners. The idea proposed in the study is that although the non-native speakers have less exposure to particular grammar structures, due to both their being non-native and their low level of education, the type of linguistic experience matters more than the sheer amount: high educated bilingual speakers have the benefit of schooling and thus show convergence to monolingual competence. This account emphasizes the role of language competence, although it does not specify what kind of metalinguistic skills are required for the processing of complex sentences.

A complementary psycholinguistics account is based on the idea of a different competition of alternative structure in bilingual speakers, with less competent speakers experiencing reduced competition between alternative structures due to a weaker knowledge of the grammar (Pickering and Branigan, 1999). This account is compatible to the linguistic account presented above, where possible differences between bilinguals and monolinguals are related to a qualitatively different encoding of the grammar due to the bilingual experience. In specific, according to this approach bilingual learning discard ambiguity and competitions between alternative interpretations, being the opportunities to speak the language confined to specific contexts, often based on a more formal use of the language and few opportunities to speak with a diverse range of speakers.

We now turn to the characteristics of Sardinian as a minority language and of bilingualism in Sardinian and Italian experienced by the participants in our study.

## Sardinian–Italian Bilingualism

Sardinian is a Romance language spoken in the Sardinian region by approximately 1.2 million people. Since 1996 it is officially recognized, together with Italian, as an official language of the island and protected by Italian laws as a minority language (Italian republic Law 482/1999 and Sardinian regional Law 26). Both laws were introduced to support the use of the minority language in schools and to promote its use in official documents of use in administration. The use of Sardinian in the public administration was supported by the promotion of an official written standard system, the adoption of which has generated a controversial debate.

The term Sardinian language (or *Limba Sarda* in Sardinian) refers to all varieties spoken in the island. Participants involved in this study are proficient in the variety spoken in the Nuoro Province, which is located in the center of the island, as can be seen in the map below.

Most Sardinians regard themselves as bilingual. According to recent extensive surveys on the languages spoken in the island (Ingrassia, 2007; Oppo, 2007), less than 3% of participants reported not to speak Sardinian, or one of its varieties. Around 68% is fluent in both comprehension and production and the remaining 29% are “passive bilinguals” who can understand the language but do not speak it. It is interesting to note for the purpose of the present research that the distribution of Sardinian speakers radically changes according to age, with a marked drop in speaker numbers among the new generations. While Oppo’s study reported a large number of bilinguals (around 85%) in the older population, the situation is different for younger adults, with percentages around 59% in the 25–45 years age band. Education is also correlated with the Sardinian/Italian bilingual status: the near totality of people with lower educational level reporting knowledge of Sardinian (95% for a primary degree and 75% for people with a secondary degree), whereas only 55% of people with a university degree report knowledge of Sardinian. A difference in the use of the language also emerges if one compares small rural towns, where Sardinian is more widely spoken, and larger towns, where Italian is the most common language used.

Oppo’s study points to the fact that Sardinian, like many other regional minority languages, is declining due to the lack of intergenerational transmission (see Romaine, 2007; Extra and Gorter, 2008). Fewer and fewer parents speak the minority language to their children because of its perceived lack of “usefulness” or the possibility of a damaging effect on Italian as the dominant language and the only language of schooling. Although Sardinian has a considerable number of speakers compared to other minority languages and it is often described as integral part of the cultural identity of Sardinians, it is not taught a school or included in any medium education program.

### Sardinian Grammar

Sardinian is a Romance language with relatively free word order, with SVO perceived as the unmarked order [see Jones (1993) and Pittau (1991) for more details]. It has a rich inflectional system and a full pronominal system characterized by a consistent use of clitic pronouns. Sardinian, like Italian, is a pro-drop language which allows omission of subject pronouns; the choice between pronoun omission and overt pronoun realization is governed by pragmatic and stylistic factors, whereas objects pronouns are obligatorily realized. Pronouns are inflected for case but case is not used in verbal inflection.

Regarding the structures included in this study and shown in **Table 1** above, passive structures are very rarely attested and they are perceived as due to transfer from Italian. This is also the case of object relative clauses, not attested in spontaneous speech; the preferred structure is a passive object relative instead, as in (5).

(5) Su pitzineddu chi l’est sighinde su cane est pompiande sa pitzinna.

The boy that he is chased by the dog is looking at the girl.

More common is the use of a resumptive pronoun in relative clauses, which is not an option in standard Italian.

## Research Question

This study aims to investigate whether Sardinian–Italian bilingual adults have a disadvantage compared with monolingual Italians in their comprehension of Italian due to interference from the minority language, and, if they do, whether the disadvantage is restricted to comprehension of more complex sentences.

Furthermore, the study aimed to explore whether there is a difference between bilingual and monolingual speakers due to level of education, since a higher educational level entails more extensive use of Italian at the expense of Sardinian, and therefore less active bilingualism.

The following questions were addressed:

(a) Do Sardinian/Italian bilingual adults have a disadvantage in Italian sentence comprehension compared to Italian monolinguals, and in particular in more complex sentences?
(b) Do Sardinian/Italian bilingual adults have an advantage in cognitive abilities related to executive function and working memory, compared to monolinguals?
(c) Are bilinguals with higher levels of education more similar to monolinguals, compared to bilingual with lower education levels, due to a less active and qualitatively different bilingual experience?

## Materials and Methods

### Participants

Sixty-three adults (mean age: 39 years; *SD*: 6.5; age range 28–50 years; 36 females) were included in the study. They all lived in the Nuoro Province in Sardinia, where Italian is the dominant language but Sardinian is also widely spoken, especially in small towns. All Sardinian–Italian speakers included in the study (*N* = 34; mean age 39.7 years; *SD*: 6.51) came from villages with no more than 9000 inhabitants; monolinguals (*N* = 29; mean age 38.6 years; *SD*: 6.64) were recruited from Nuoro, Macomer, and Tortoli, towns with more than 9000 inhabitants. This condition mirrors the general distribution of Sardinian speakers in the island, with bilingual Sardinian–Italian speakers living in more rural areas and monolingual Italian speakers living in the larger towns (Oppo, 2007).

Bilingualism was measured using the Bilingual Language Profile (BLP) scale (Birdsong et al., 2012). This questionnaire consists of 19 questions and focuses on different aspects of the participant’s language experience in both the dominant and the minority language. An overall score given by the average scores on all four measures of the two languages (Sardinian and Italian), the amount of exposure to each language measured with six questions exploring age of acquisition and years of language learning in different contexts (school, family, work, friends, country), the overall use of each language measured as the percentage of time speaking the language in different contexts (speaking with friends, in the family, at work, with himself, and use for counting), the competence (a ranking self-assessment on production, comprehension, reading, and writing), and the attitude toward the minority language (four ranked questions on the speakers degree of identification as a speaker of the language) were recorded for all participants^3^.

Education in Sardinia is only through the medium of Italian; we recorded the level of education for all participants by asking if they stopped after secondary school (SEC: secondary school degree) or if they had a university degree (UNI: graduate participants with a university degree). Four participants with only primary school education were excluded from the sample, as well as one participant with an outlier performance on one of the test measures (overall *Comprendo* timing greater than 3 SD). See summary in **Table 2** below.

**Table 2:** Bilingual language profile (BLP) average scores for both languages, namely Sardinian and Italian for the four groups: bilinguals with secondary education, monolinguals with secondary education, bilinguals with university degree, and monolinguals with university degree.

	Group; total *N* = 63	Overall score (means average of four factors)	Exposure	Use	Competence	Attitude
Sardinian	Bilinguals SEC (15)	164.4	91.9	30.5	17.1	22.3
	Bilinguals UNI (19)	165.3	82.9	28.8	18.9	23.5
	Monolinguals SEC (18)	39.9	43.6	0.1	4.6	4.3
	Monolingual UNI (11)	33.8	23.8	0.1	3.3	6.8
		**Overall**	**Exposure**	**BLP use**	**Competence**	**Attitude**
Italian	Bilinguals SEC (15)	145.6	91.1	19.5	21.1	15.5
	Bilinguals UNI (19)	159.2	100.6	21.2	22.9	16.9
	Monolinguals SEC (18)	205.5	107.2	49.9	22.5	22.6
	Monolingual UNI (11)	213.1	113.3	49.3	23.7	23.8


*BLP: Bilingual Language Profile (Birdsong et al., 2012). SEC: participants with secondary school degree; UNI: participants with university degree. OVERALL SCORE: average score for the four measures (exposure, use, competence, and attitude). EXPOSURE: average score for five questions on age of acquisition and number of years of exposure in different contexts (0–100%). USE: average score for five questions on language use in different contexts (0–100%). COMPETENCE: average score on four questions on language competence. ATTITUDE: average score on attitudes and degree of identification as speaker of the language.*

It is interesting to note that exposure, use, competence, and attitude toward Sardinian clearly distinguish between monolinguals and bilinguals: comparisons on all BLP dimensions are highly significant (Sardinian use: *t* = 13.0; Sardinian comprehension: *t* = 13.1; attitude toward Sardinian, *t* = 15.4; all *p* < 0.001). Only exposure to Sardinian shows a difference in education as well, with *t*(group) = 11.5 and *t*(education) = 3.7; both *p* < 0.001. For these BLP measures, bilinguals with lower level of education reported higher exposure to Sardinian compared to all other groups.

As for the BLP Italian dominance measures, while the group differences are less marked than in the Sardinian BLP measures, they are nevertheless significant [exposure to Italian, *t*(group) = 4.48, *p* < 0.001 and *t*(education) = 2.45, *p* = 0.017; Italian usage *t*(group) = 13.29, *p* < 0.001; Italian comprehension, *t*(group) = 3.40, *p* = 0.00122 and *t*(education) = 2.50, *p* = 0.1531; attitude toward Italian, *t*(group) = 4.96, *p* < 0.001]. The distribution of Italian competence for the four groups recorded with the BLP is shown in **Figure 2**.

### Test Measures

#### Comprendo

**FIGURE 1:**
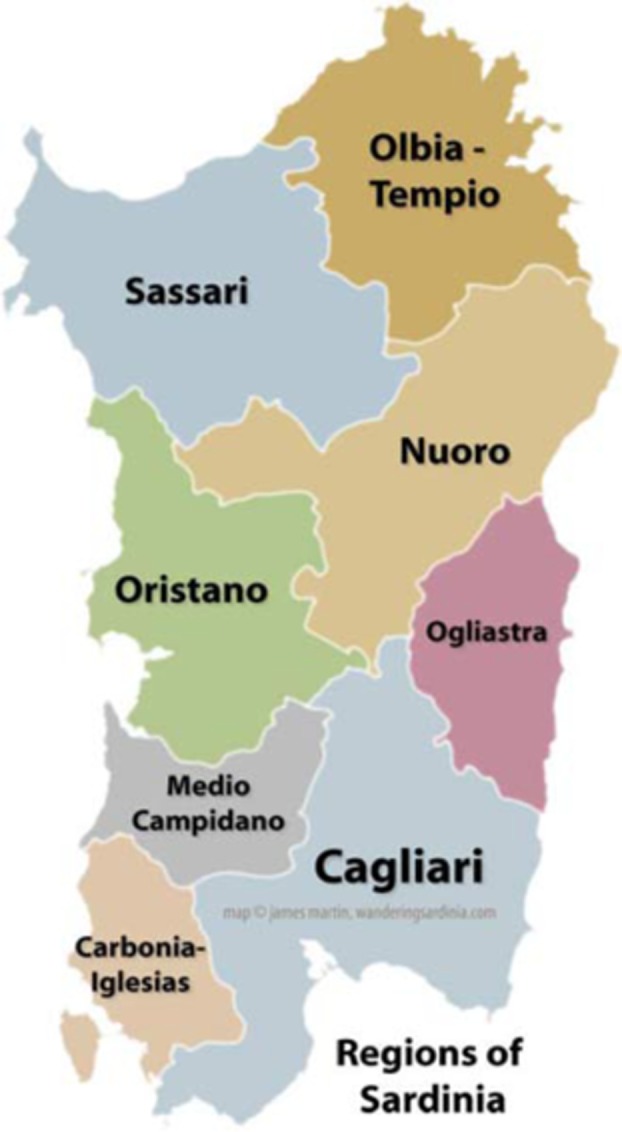
Map of Sardinian Provinces.

**FIGURE 2:**
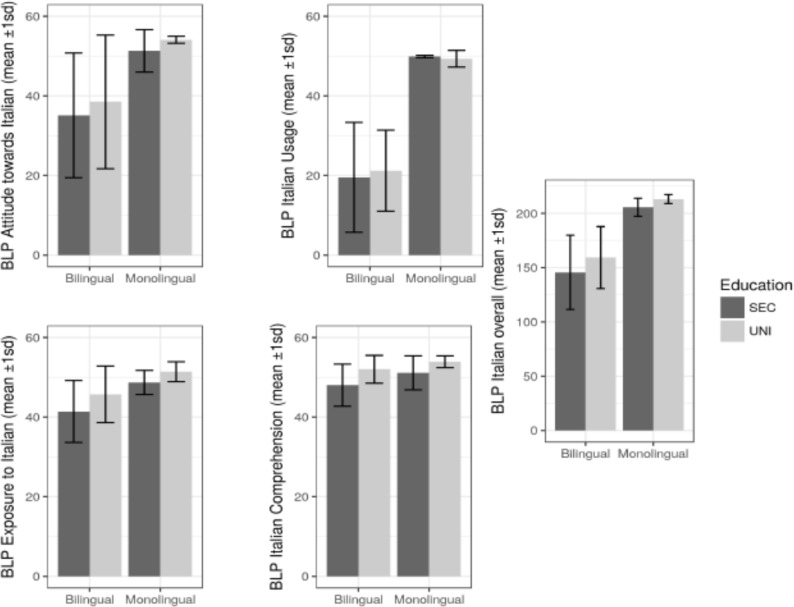
BLP results for Italian for all four groups (Bilingual SEC, Bilingual UNI, Monolingual SEC, and Monolingual UNI): Overall, Exposure, Usage, Competence, and Attitude.

We focused on language comprehension of the dominant language, Italian, by testing the comprehension of sentences with different degrees of grammatical complexity in order to establish whether there were differences in Italian competence due to bilingualism, and whether these differences were less marked in the bilingual group with a higher education level and more use of Italian. We used *Comprendo* (Cecchetto et al., 2012), a comprehensive test battery developed for recording both accuracy and reaction times (RTs) in adults. The battery includes a range of sentences differing in complexity, from simple active sentences to more complex relative clauses, across 10 different sentences types (see **Table 1** in the section “Linguistic Abilities and Knowledge of Grammar in Bilingualism”). There were 10 items per condition, with a total of 100 items per *Comprendo* trial. For each sentence, the participant was asked to select one of four pictures (see example in **Figure 1**). The correct picture matched the sentence meaning: for the sentence “La mamma da la torta al bambino” (*The mum gives the cake to the boy*), the correct picture showed a mother giving a cake to a young boy (D in **Figure 2**). In addition, there were three incorrect “distractor” pictures. The *reversal distractor* depicted the same actors in reversed roles (e.g., a boy giving a cake to his mother). The *verbal distractor* depicted the actors in the same thematic roles, but completing a different action (e.g., the mother caressing the boy). The *nominal distractor* kept the same action (e.g., giving), but replaced all the nouns (both the actors and the object; e.g., *The grandmother gives the keys to the girl*). The task requires mapping the thematic roles (i.e., *Who is doing what to whom?*) in relation to the syntactic form of the sentence.

**FIGURE 3:**
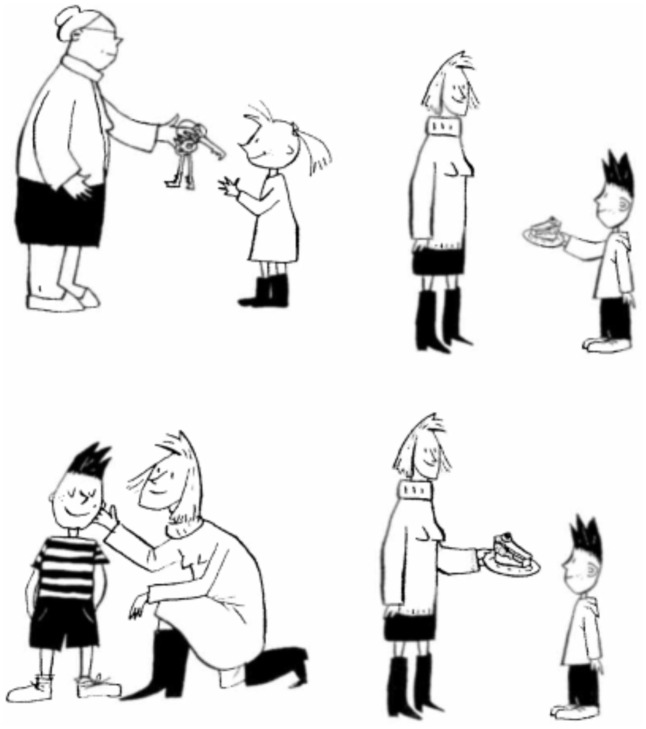
*Comprendo* sample pictures for the dative target sentence “La mamma dà la torta al bambino” (*The mum gives the cake to the child*). Correct picture in the bottom right quadrant.

Both accuracy and RTs were recorded via E-Prime. Subjects heard a sentence pre-recorded on a laptop and 1000 ms before the end of the recording, a picture, as the one in **Figure 3**, was displayed on the screen for a fixed time of 300 ms. This procedure, adopted from a previous study (Lauro et al., 2010), allowed a uniform onset of the picture across trials even though the length of the recording was variable (due to the different number of words for each sentence). Subjects were asked to select which picture represented the meaning of the sentence by pressing a response key (this measure capture both accuracy and RTs). The task was run in one session, with sentences presented in random order.

#### Backward Digit Span

Working memory was therefore assessed using a backward digit span test adapted from Orsini et al. (1987). The experimenter read aloud a string of digits, at the pace of one second per digit, and the participant had to repeat the digits backward. Each sequence was incrementally longer. A sequence was considered correct if the participant repeated the whole sequence in the right backward order. There were two strings of digits per length. A score consisted of the longest sequence repeated correctly.

#### BCOS Rule Finding and Concept Switching Test

The rule finding and concept-switching test is a visual task aiming at detecting the ability to switch rule after inferring it. It is a measure of non-verbal intelligence, designed to be used with patients with language impairments and it is part of a larger battery for evaluating cognitive impairment in people with brain injuries (Humphreys et al., 2012). It consists of a set of cards with a grid and colored dots spaced on the grid. The participants have to infer where the black dot will appear in the next card avoiding the interference of the currently active rule. The test measures both correct responses and correct rules inferred in a unique score.

#### Stroop Task

We adopted the Italian version of the Stroop test: *Test di Stroop*, *versione breve* developed by Valgimigli et al. (2010). After a non-timed practice trial on both congruent and incongruent conditions, the participant starts by naming colors in the congruent condition and then in the non-congruent conditions. Participants have 20 s for each condition (congruent and incongruent naming). Each condition has a maximum of 60 items. In both conditions, correctly named items in 20 s are recorded. The Stroop effect is calculated according to the following formula: Interference effect = [(Named colors/ Congruent – Named colors/Incongruent)/(Named colors/ Congruent + Named colors/Incongruent)] ^∗^ 100. Low scores are an index of low interference levels.

### Statistical Analysis

Linear regression models were used to test for significant differences across the four groups (monolingual vs. bilingual by two education levels) in the various test measures described above. Additionally, for the Comprendo sentence-to-picture matching tasks, linear mixed effects regression models were run to test for timing differences across the 10 sentence types. This is because the picture choices with different sentence types typically take different amount of time to carry out, and hence times were included in the mixed effect models as random intercepts.

## Results

### Comprendo

The average correct response (out of a maximum 100) across all groups was 90.2 (*SD* = 5.24), in line with what has been reported for standardized assessment scores (Cecchetto et al., 2012). Overall means for RTs were 4375 ms (*SD* = 700). Both the overall bilingual RTs and the overall monolingual RTs matched the overall time ranges reported in the standard assessment for the 40–49 years age range (bilingual mean was 4193, vs. 4242 for the standardized test; monolingual mean was 4589 ms, vs. 4242 for the standardized test).

In terms of accuracy (number of correct responses), there were no significant differences between monolinguals and bilinguals, across the two education levels. Monolinguals adults with a secondary education scored the lowest (AM SEC mean 87.0, *SD*: 6.81), and monolingual adults with a university education scored the highest (AM UNI mean: 93.1, *SD*: 3.08).

As for sentence complexity, adult monolinguals with a lower education level tended to take longer than adult bilinguals with a lower education level in processing center embedded s*ubject* relative clauses (AB.SEC *m*(sd) = 4613(1129) s, AM.SEC *m*(sd) = 5240(1009) s *t*(31) = -1.68, *p* = 0.102, two-tailed) and o*bject* relative clauses (AB.SEC *m*(sd) = 4556(871) s, AM.SEC *m*(sd) = 5293(773) s, *t*(31) = -2.58, *p* = 0.015, two-tailed). In **Table 3** RTs for all sentences types are reported.

**Table 3:** Mean timing (ms) for each *Comprendo* sentence types across the two education levels and the two language groups.

Sentences	Bilingual SEC	Bilingual UNI	Monolingual SEC	Monolingual UNI
Active	3585	3292	3657	3640
Dative	4098	4027	4307	4143
Passive	3741	3514	3973	3656
Relative subject peripheral	3296	3208	3589	3488
Relative object peripheral	4435	4369	4683	4460
Relative subject center	4424	4300	5107	4661
Relative object center	4556	4554	5293	4616
Object coordination	5182	4521	5366	5350
Verb Coordination	5566	4685	5677	5963
Sentence coordination	4358	4247	4568	4404


**FIGURE 4:**
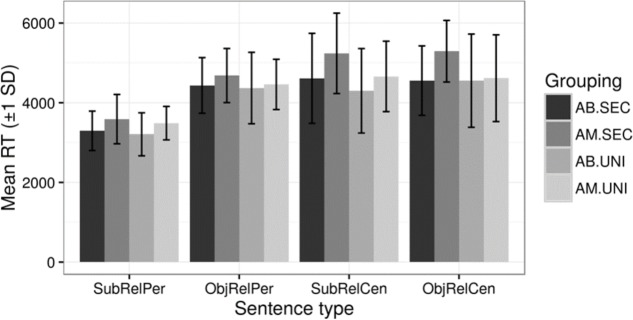
Mean of RTs for comprehension of relative clauses across the four groups.

The variation among participants in the time taken for subject relative clauses was relatively large, as can be seen in **Figure 4**.

**FIGURE 5:**
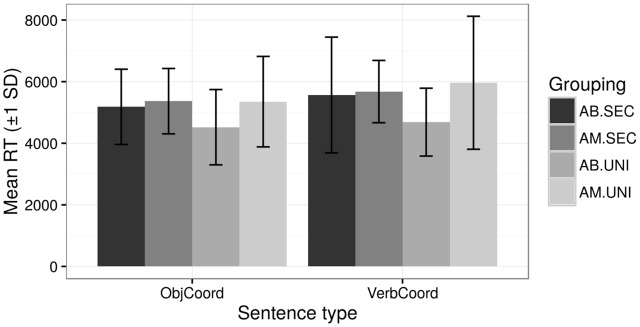
Mean RTs for comprehension of long sentences with simple syntax in the four groups.

As shown in **Figure 5** below, adult bilinguals with university education (AB UNI) took less time than their similarly educated monolingual counterparts (AM UNI) in the two conditions with longest sentences measured in term of number of word, namely object coordination and verb coordination. These sentences are longer with no syntactic complexity. However, there was again much variation among participants and the differences only approach significance: for the object coordination clauses, AB.UNI *m*(sd) = 4521(1222) s, AM.UNI *m*(sd) = 5350(1468) s, *t*(28) = -1.66, *p* = 0.107 two-tailed; for the verb coordination clauses, AB.UNI *m*(sd) = 4685(1101) s, AM.UNI *m*(sd) = 5962(2159) s, *t*(28) = -2.16, *p* = 0.034 two-tailed.

The distribution of the scores on the backward digit span reveals that four bilinguals (11.8%) had the higher score (span of 6) compared to only one participant (3.5%) in the monolingual group. In particular, the monolingual secondary-educated group (AM SEC) appears to perform worse than the other three groups (mean = 4.11). Likewise, the bilingual university-educated group (AB UNI) is the more proficient of the four groups (mean = 4.58). Additionally, there appears to be an effect related to the bilingual experience: monolingual secondary-educated participants performed worse than the bilingual secondary-educated participants (AB.SEC *m* = 4.40). The performance on the backward digit span will be discussed below in relation to the RTs results from the Comprendo test.

### Stroop Task

The Stroop task showed only a tendency for those with a university education to have smaller interference effects (intercept mean STROOP interference for UNI: 17.5; estimate difference for SEC: 2.88, SE = 1.7, *t* = 1.68, *p* = 0.098) but there were no interactions with language background.

### B-COS Task

None of the three B-COS task measures showed any differences between language groups or education levels. The overall mean (SD) were: N60 = 0.57(0.946); N18 = 12.2(2.73); N3 = 2.27(0.632).

## Discussion

In this study, we compared the performance of a group of adult Sardinian/Italian speakers with low education (secondary school) with a group of monolinguals with the same educational level and living in the same area of Sardinia. To better control for the impact of the educational level, a similar group of bilinguals with a university degree was compared with a group of monolinguals with the same educational level.

All groups performed close to ceiling in all conditions tested, including the tasks that measured cognitive control and working memory. Although performance on the accuracy of sentence comprehension, measured with the *Comprendo* test, did not differentiate between monolinguals and bilinguals, the RTs in this task revealed an interesting difference in the processing of complex sentences, which was slower for monolinguals with lower education compared to bilinguals with a similar education level.

Monolingual Italian speakers with a low level of education (AM SEC in this study) represent the group with the slowest performance in the sentence comprehension task. Bilingual Sardinian/Italian speakers with lower education (AB SEC) were faster than monolinguals, this was significant during comprehension of complex center embedded object relative clauses in Italian. No difference in processing costs was found between the two groups with university education: this is arguably due to the effect of more intensive use of Italian, which levels off any differences due to bilingualism. Bilingual speakers with high education reported low level of use of the minority languages; this is because Italian is the dominant and often only language used in high-educated environments.

The faster processing found for less educated bilingual speakers suggests that the active use of the minority language has a *positive* impact on language competence in the dominant language, which partly compensates the effects of low education levels. Looking back at their language profile (collected with the BLP), bilinguals with secondary education in fact reported higher use of Sardinian compared to monolinguals and consequently less use of Italian. Also interesting is the selective effect on comprehension of object relatives, which shows a faster performance in the bilingual group with lower education compared to monolinguals. It is possible that the use of Italian in the group of bilinguals is associated with a more restricted range of linguistics contexts, which results in less competition among alternative syntactic structures, and consequent faster processing for complex sentences. One option is that bilingual speakers, with low use of Italian and only in formal contexts, have a linguistically different competence in the dominant language and it will be not natural for them to retrieve less costly but alternative grammatical structures. Avoidance strategies based on preference of less costly grammatical structures, as in the case of a preference for passive object relatives instead of object relatives, are often reported in studies on monolinguals (Belletti and Guasti, 2015). It is interesting to note that the passive object relative is the natural option for Sardinian speakers in the context of object relatives (see **Table 1** above). But it is possible that the two languages are segregated in their use to two difference registers, richer for Sardinian and more formal for Italian.

A better performance on complex sentences in non-native speakers, similar to our study, was reported in studies on L2 learners; Dubrowska and Street (2006), in a study on comprehension of passive sentences in L2 learners found that less-educated non-native speakers perform better than native speakers English speakers. This converging evidence supports the idea of a linguistically based difference in bilinguals compared to monolinguals with an advantage in the comprehension of more complex sentences in bilinguals due to a different grammatical representation for these sentences.

A second finding of our study is the enhanced performance of adult bilingual speakers on the working memory task. This group showed better results in the Digit Span task compared to monolinguals: 11% of the adult bilinguals obtained the highest score, compared to only 3% of the adult monolingual sample. Considering that both groups are living in a similar setting, it is plausible that the enhancement in working memory may be related to the bilingualism of the Sardinian/Italian group. Interestingly this group does not show any difference in grammatical processing of Italian, where their performance converges with that of monolinguals, but they possibly show the effects of the bilingual experience in their faster processing of long sentences with no greater complexity. This finding supports the cognitively based model, with better working memory skills in bilinguals compared to monolinguals, as reported in other studies on cognition in bilingual speakers (Bialystok, 2007). It is worth pointing out that Sardinian, as many minority languages, is used mainly orally. Many societies do not have an active written system (Montrul, 2008), and this is often the case in regional minority languages, where communications needs are shifted toward an orality. In this study the group of low educated bilinguals showed better working memory scores compared to monolingual speakers with university-level education. This result is not explainable by standard model of WM, where WM is often related to higher levels of education (Murre et al., 2013). Future research it is necessary to address the question of whether the effects of bilingualism are modulated by the modality of language use, for example focusing more on the effects of the exclusive oral use of a language^4^.

## Conclusion

This study focused on the Sardinian/Italian bilingualism in the Nuoro Province, the area of Sardinia where the minority language is well used and preserved. A previous study on Sardinian/Italian children living in this area (Garraffa et al., 2015) reported similar comprehension of Italian in bilingual and monolingual children starting primary school in the Nuoro Province, suggesting that the minority language does not interfere negatively with the development of the majority language and that some beneficial cognitive effects emerge gradually over time in bilingual children speaking Sardinian. In extending the investigation of Sardinian/Italian bilingualism to speakers in the adult community, our study again found no differences in comprehension of Italian, and in addition some advantages in memory skills and faster processing of more complex sentences in Sardinian/Italian bilinguals. Both studies supported the idea that minority languages can be beneficial for language competence and some aspects of cognition, although more research is needed to explore the exact source of the differences in processing complex sentences and the mutual effects of cognitive and linguistics capacities in bilingual speakers of minority languages.

## Ethics Statement

All subjects gave written informed consent in accordance with the Declaration of Helsinki. The protocol was approved by the “University of Edinburgh, School of PPLS Ethic committee.”

## Author Contributions

MG: conception and design of the work; analysis and interpretation of data for the work; drafting the work and revising it critically for important intellectual content; final approval of the version to be published; agreement to be accountable for all aspects of the work in ensuring that questions related to the accuracy or integrity of any part to the work are appropriately investigated and resolved. MO: analysis and interpretation of data for the work; drafting the work and revising it critically for important intellectual content; agreement to be accountable for all aspects of the work in ensuring that questions related to the accuracy or integrity of any part of the work are appropriately investigated and resolved. AS: conception and design of the work; interpretation of data for the work; drafting the work and revising it critically for important intellectual content; final approval of the version to be published; agreement to be accountable for all aspects of the work in ensuring that questions related to the accuracy or integrity of any part of the work are appropriately investigated and resolved.

## Conflict of Interest Statement

The authors declare that the research was conducted in the absence of any commercial or financial relationships that could be construed as a potential conflict of interest.

## Footnotes

1 In particular in the case of object relatives, the element extracted from the relative clauses (in italics) cross a potential candidate for the same position (in bold), making this structure more complex due to grammatical interference [see Garraffa and Grillo (2008), Grillo (2008) and Rizzi (2013) for a detailed description of interference effects in object relatives].

2 It is possible that the effect on the dominant language of bilingualism in another language is visible both in production and in comprehension due to the necessary involvement of a production step for comprehension of complex sentences, as predicted by working memory-based models [see also Riches and Garraffa (2017) for an overview of grammatical-based effects in children and adults].

3 For a detailed description of the BLP Questionnaire and all its items, see https://sites.la.utexas.edu/bilingual/. All materials are available online.

4 An argument in favor of the impact of oral use on memory was proposed in Plato’s Phaedrus in the *Myth of Theuth*, *the God of writing* (Plato, 360 B.C., 274b–279b). Socrates in a well-known passage commented on the invention of writing as follows: “it will create forgetfulness in the learners’ souls, because they will not use their memories; they will trust to the external written characters and not remember of themselves. The specific which you have discovered is an aid not to memory, but to reminiscence.”
